# Supplementary figures and images for: The evaluation of the utility of the GENECUBE HQ SARS-CoV-2 for anterior nasal samples and saliva samples with a new rapid examination protocol
Source: PLoS One. 2021 Dec 31;16(12):e0262159. doi: 10.1371/journal.pone.0262159 (PMC8719657; doi:10.1371/journal.pone.0262159)

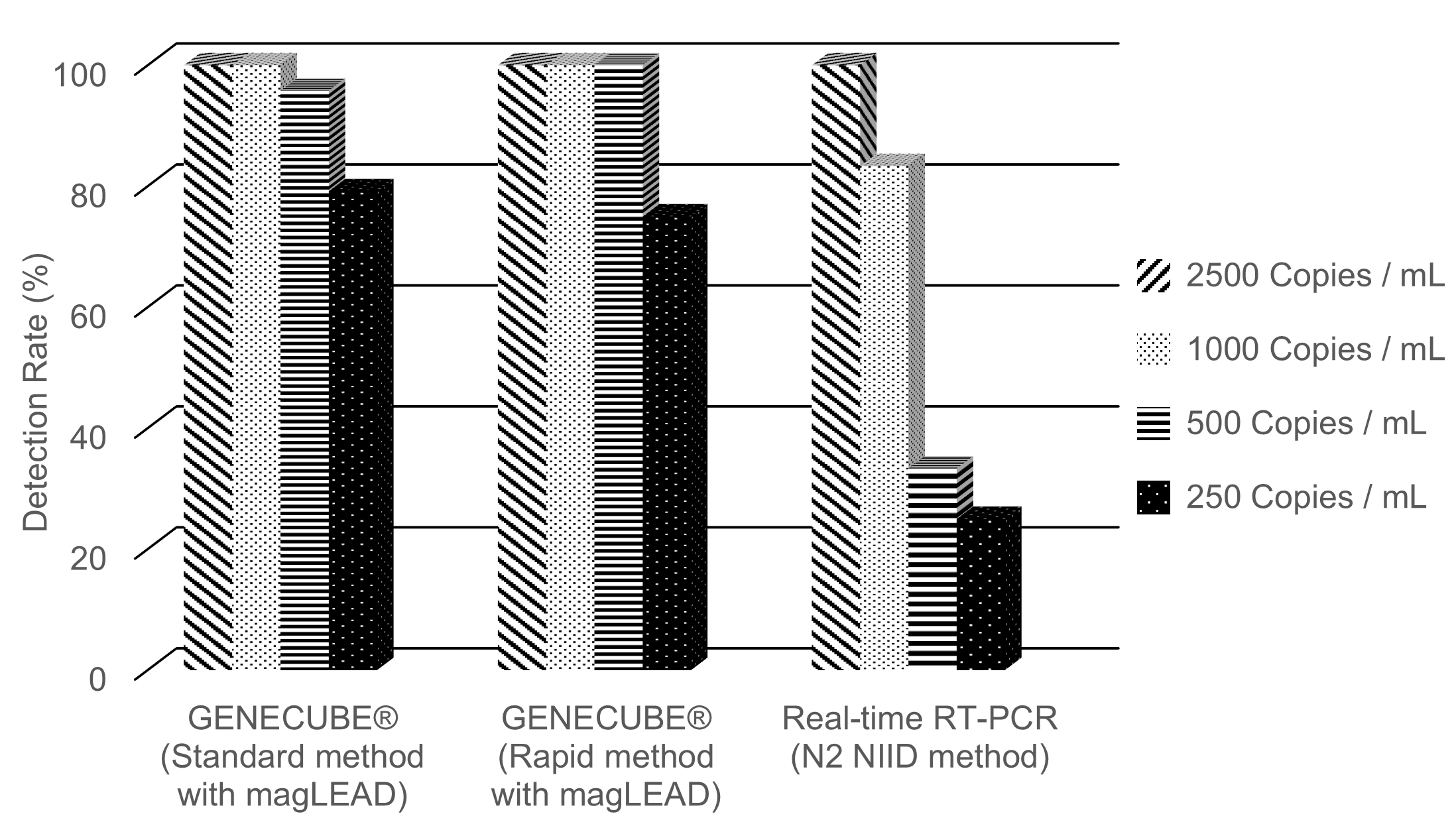

Supplement: S1 Fig — The same set of samples was extracted with the standard or rapid method with magLEAD and analyzed with GENECUBE® and real-time RT-PCR (N2 NIID method) for each sample concentration. The vertical axis shows the detection rate (%). The horizontal axis shows the comparison of each three SARS-CoV-2 detection methods, and each of the bar graph types shows the sample concentration. (TIF) [file pone.0262159.s001.tif]
