# Supplementary material for: The evaluation of the utility of the GENECUBE HQ SARS-CoV-2 for anterior nasal samples and saliva samples with a new rapid examination protocol
Source: PLoS One. 2021 Dec 31;16(12):e0262159. doi: 10.1371/journal.pone.0262159 (PMC8719657; doi:10.1371/journal.pone.0262159)
Supplement: S1 Table — (DOCX) [file pone.0262159.s002.docx]

**S1 Table. Results of SARS-Cov-2 detection for anterior nasal samples.**

| Anterior nasal samples | | | | | Nasopharyngeal samples collected from the same patient simultaneously | | |
| --- | --- | --- | --- | --- | --- | --- | --- |
| Sample No. | GENECUBE_®_  (Standard method with magLEAD) | Real-time RT-PCR  (N2 NIID method) | | | GENECUBE_®_  (Standard method with magLEAD) | Real-time RT-PCR  (N2 NIID method) | |
|  |  | Ct | Ct | Copies/test |  | Ct | Copies/test |
| #1 | + | 18.1 | 18.1 | 171,250 | + | 13.7 | 3,828,000 |
| #2 | + | 20.3 | 20.3 | 99,765 | + | 11.3 | 18,800,000 |
| #3 | + | 22.8 | 22.8 | 18,340 | + | 19.1 | 145,600 |
| #4 | + | 29.8 | 29.7 | 162 | + | 16.5 | 745,000 |
| #5 | + | 37.7 | ND | 1 | + | 24.5 | 5,063 |
| #6 | + | 22.3 | 22.3 | 24,840 | + | 14.1 | 7,360,000 |
| #7 | + | 24.1 | 24.1 | 7,601 | + | 17.8 | 588,500 |
| #8 | + | 23.3 | 23.3 | 9,821 | + | 13.2 | 4,672,000 |
| #9 | + | 23.1 | 23.2 | 10,725 | + | 16.5 | 601,000 |
| #10 | + | 19.9 | 19.9 | 202,700 | + | 20.0 | 103,400 |
| #11 | + | 24.6 | 24.6 | 6,764 | + | 25.0 | 3,959 |
| #12 | + | 18.6 | 18.6 | 753,200 | + | 16.4 | 3,162,000 |
| #13 | + | 14.8 | 14.8 | 6,096,500 | + | 15.4 | 7,636,000 |
| #14 | + | 16.6 | 16.5 | 1,804,500 | + | 17.4 | 1,569,000 |
| #15 | + | 33.1 | 32.6 | 31 | + | 31.1 | 68 |
| #16 | + | 17.8 | 17.8 | 707,300 | + | 13.3 | 11,340,000 |
| #17 | + | 26.4 | 26.4 | 1,810 | + | 19.9 | 135,000 |
| #18 | + | 18.1 | 18.1 | 574,000 | + | 16.5 | 1,258,000 |
| #19 | + | 20.9 | 20.9 | 41,260 | + | 13.0 | 5,015,000 |
| #20 | + | 34.2 | 34.3 | 10 | + | 29.1 | 283 |
| #21 | + | 31.1 | 31.1 | 76 | + | 25.7 | 2,654 |
| #22 | + | 18.4 | 18.4 | 268,950 | + | 19.3 | 151,000 |
| #23 | + | 19.9 | 19.9 | 102,100 | + | 20.5 | 72,690 |
| #24 | + | 16.3 | 16.3 | 3,603,000 | + | 14.0 | 19,650,000 |
| #25 | + | 18.5 | 18.5 | 303,200 | + | 15.8 | 1,734,000 |
| #26 | + | 19.5 | 19.5 | 154,100 | + | 17.8 | 472,300 |
| #27 | + | 27.1 | 27.2 | 818 | + | 19.1 | 106,000 |
| #28 | + | 34.2 | 33.8 | 14 | + | 24.8 | 3,465 |
| #29 | + | 29.7 | 29.8 | 172 | + | 24.3 | 7,297 |
| #30 | + | 21.6 | 21.5 | 49,900 | + | 19.3 | 236,400 |
| #31 | + | ND | 37.7 | 1 | + | 22.8 | 14,010 |
| #32 | + | 16.7 | 16.8 | 605,850 | + | 14.7 | 2,212,000 |
| #33 | + | 25.9 | 26.0 | 2,380 | + | 23.4 | 13,770 |
| #34 | + | 16.8 | 16.8 | 1,257,500 | + | 15.5 | 2,982,000 |
| #35 | + | 23.1 | 23.2 | 12,155 | + | 15.6 | 1,466,000 |
| #36 | + | 18.8 | 18.8 | 189,600 | + | 15.2 | 1,939,000 |
| #37 | + | 24.2 | 24.2 | 6,399 | + | 17.6 | 408,900 |
| #38 | + | 26.7 | 26.8 | 1,356 | + | 17.4 | 863,300 |
| #39 | + | 25.5 | 25.5 | 3,146 | + | 17.3 | 641,800 |
| #40 | + | 25.5 | 25.5 | 3,239 | + | 14.1 | 5,296,000 |
| #41 | + | 20.7 | 20.8 | 70,325 | + | 16.5 | 1,102,000 |
| #42 | - | ND | 36.8 | 1 | + | 31.2 | 76 |
| #43 | + | 25.3 | 25.3 | 4,674 | + | 16.6 | 1,999,000 |
| #44 | + | 27.1 | 27.2 | 1,274 | + | 19.7 | 224,400 |
| #45 | + | 19.0 | 19.0 | 383,000 | + | 12.4 | 38,160,000 |
| #46 | + | 29.2 | 29.4 | 301 | + | 22.8 | 25,310 |
| #47 | + | 18.9 | 18.9 | 366,500 | + | 18.3 | 537,900 |
| #48 | + | 25.4 | 25.3 | 1,869 | + | 16.3 | 233,500 |
| #49 | + | 27.8 | 27.9 | 507 | + | 18.9 | 57,760 |
| #50 | + | 30.5 | 30.6 | 119 | + | 23.4 | 5,236 |
| #51 | + | 22.2 | 22.2 | 31,420 | + | 15.0 | 4,452,000 |
| #52 | + | 33.4 | 32.8 | 19 | + | 20.8 | 81,770 |
| #53 | - | ND | ND | ND | + | 20.0 | 83,610 |
| #54 | - | ND | ND | ND | + | 19.7 | 171,900 |
| #55 | - | ND | ND | ND | + | 31.7 | 54 |
| #56 | - | ND | ND | ND | + | 16.1 | 1,402,000 |
| #57 | - | ND | ND | ND | + | 19.8 | 200,500 |
| #58 | - | ND | ND | ND | + | 29.4 | 220 |
| #59 | - | ND | ND | ND | + | 26.1 | 1,281 |
| #60 | - | ND | ND | ND | - | ND | ND |
| #61 | - | ND | ND | ND | - | ND | ND |
| #62 | - | ND | ND | ND | - | ND | ND |
| #63 | - | ND | ND | ND | - | ND | ND |
| #64 | - | ND | ND | ND | - | ND | ND |
| #65 | - | ND | ND | ND | - | ND | ND |
| #66 | - | ND | ND | ND | - | ND | ND |
| #67 | - | ND | ND | ND | - | ND | ND |
| #68 | - | ND | ND | ND | - | ND | ND |
| #69 | - | ND | ND | ND | - | ND | ND |
| #70 | - | ND | ND | ND | - | ND | ND |
| #71 | - | ND | ND | ND | - | ND | ND |
| #72 | - | ND | ND | ND | - | ND | ND |
| #73 | - | ND | ND | ND | - | ND | ND |
| #74 | - | ND | ND | ND | - | ND | ND |
| #75 | - | ND | ND | ND | - | ND | ND |
| #76 | - | ND | ND | ND | - | ND | ND |
| #77 | - | ND | ND | ND | - | ND | ND |
| #78 | - | ND | ND | ND | - | ND | ND |
| #79 | - | ND | ND | ND | - | ND | ND |
| #80 | - | ND | ND | ND | - | ND | ND |
| #81 | - | ND | ND | ND | - | ND | ND |
| #82 | - | ND | ND | ND | - | ND | ND |
| #83 | - | ND | ND | ND | - | ND | ND |
| #84 | - | ND | ND | ND | - | ND | ND |
| #85 | - | ND | ND | ND | - | ND | ND |
| #86 | - | ND | ND | ND | - | ND | ND |
| #87 | - | ND | ND | ND | - | ND | ND |
| #88 | - | ND | ND | ND | - | ND | ND |
| #89 | - | ND | ND | ND | - | ND | ND |
| #90 | - | ND | ND | ND | - | ND | ND |
| #91 | - | ND | ND | ND | - | ND | ND |
| #92 | - | ND | ND | ND | - | ND | ND |
| #93 | - | ND | ND | ND | - | ND | ND |
| #94 | - | ND | ND | ND | - | ND | ND |
| #95 | - | ND | ND | ND | - | ND | ND |
| #96 | - | ND | ND | ND | - | ND | ND |
| #97 | - | ND | ND | ND | - | ND | ND |
| #98 | - | ND | ND | ND | - | ND | ND |
| #99 | - | ND | ND | ND | - | ND | ND |
| #100 | - | ND | ND | ND | - | ND | ND |
| #101 | - | ND | ND | ND | - | ND | ND |
| #102 | - | ND | ND | ND | - | ND | ND |
| #103 | - | ND | ND | ND | - | ND | ND |
| #104 | - | ND | ND | ND | - | ND | ND |
| #105 | - | ND | ND | ND | - | ND | ND |
| #106 | - | ND | ND | ND | - | ND | ND |
| #107 | - | ND | ND | ND | - | ND | ND |
| #108 | - | ND | ND | ND | - | ND | ND |
| #109 | - | ND | ND | ND | - | ND | ND |
| #110 | - | ND | ND | ND | - | ND | ND |
| #111 | - | ND | ND | ND | - | ND | ND |
| #112 | - | ND | ND | ND | - | ND | ND |
| #113 | - | ND | ND | ND | - | ND | ND |
| #114 | - | ND | ND | ND | - | ND | ND |
| #115 | - | ND | ND | ND | - | ND | ND |
| #116 | - | ND | ND | ND | - | ND | ND |
| #117 | - | ND | ND | ND | - | ND | ND |
| #118 | - | ND | ND | ND | - | ND | ND |
| #119 | - | ND | ND | ND | - | ND | ND |
| #120 | - | ND | ND | ND | - | ND | ND |
| #121 | - | ND | ND | ND | - | ND | ND |
| #122 | - | ND | ND | ND | - | ND | ND |
| #123 | - | ND | ND | ND | - | ND | ND |
| #124 | - | ND | ND | ND | - | ND | ND |
| #125 | - | ND | ND | ND | - | ND | ND |
| #126 | - | ND | ND | ND | - | ND | ND |
| #127 | - | ND | ND | ND | - | ND | ND |
| #128 | - | ND | ND | ND | - | ND | ND |
| #129 | - | ND | ND | ND | - | ND | ND |
| #130 | - | ND | ND | ND | - | ND | ND |
| #131 | - | ND | ND | ND | - | ND | ND |
| #132 | - | ND | ND | ND | - | ND | ND |
| #133 | - | ND | ND | ND | - | ND | ND |
| #134 | - | ND | ND | ND | - | ND | ND |
| #135 | - | ND | ND | ND | - | ND | ND |
| #136 | - | ND | ND | ND | - | ND | ND |
| #137 | - | ND | ND | ND | - | ND | ND |
| #138 | - | ND | ND | ND | - | ND | ND |
| #139 | - | ND | ND | ND | - | ND | ND |
| #140 | - | ND | ND | ND | - | ND | ND |
| #141 | - | ND | ND | ND | - | ND | ND |
| #142 | - | ND | ND | ND | - | ND | ND |
| #143 | - | ND | ND | ND | - | ND | ND |
| #144 | - | ND | ND | ND | - | ND | ND |
| #145 | - | ND | ND | ND | - | ND | ND |
| #146 | - | ND | ND | ND | - | ND | ND |
| #147 | - | ND | ND | ND | - | ND | ND |
| #148 | - | ND | ND | ND | - | ND | ND |
| #149 | - | ND | ND | ND | - | ND | ND |
| #150 | - | ND | ND | ND | - | ND | ND |
| #151 | - | ND | ND | ND | - | ND | ND |
| #152 | - | ND | ND | ND | - | ND | ND |
| #153 | - | ND | ND | ND | - | ND | ND |
| #154 | - | ND | ND | ND | - | ND | ND |
| #155 | - | ND | ND | ND | - | ND | ND |
| #156 | - | ND | ND | ND | - | ND | ND |
| #157 | - | ND | ND | ND | - | ND | ND |
| #158 | - | ND | ND | ND | - | ND | ND |
| #159 | - | ND | ND | ND | - | ND | ND |
| #160 | - | ND | ND | ND | - | ND | ND |
| #161 | - | ND | ND | ND | - | ND | ND |
| #162 | - | ND | ND | ND | - | ND | ND |
| #163 | - | ND | ND | ND | - | ND | ND |
| #164 | - | ND | ND | ND | - | ND | ND |
| #165 | - | ND | ND | ND | - | ND | ND |
| #166 | - | ND | ND | ND | - | ND | ND |
| #167 | - | ND | ND | ND | - | ND | ND |
| #168 | - | ND | ND | ND | - | ND | ND |
| #169 | - | ND | ND | ND | - | ND | ND |
| #170 | - | ND | ND | ND | - | ND | ND |
| #171 | - | ND | ND | ND | - | ND | ND |
| #172 | - | ND | ND | ND | - | ND | ND |
| #173 | - | ND | ND | ND | - | ND | ND |
| #174 | - | ND | ND | ND | - | ND | ND |
| #175 | - | ND | ND | ND | - | ND | ND |
| #176 | - | ND | ND | ND | - | ND | ND |
| #177 | - | ND | ND | ND | - | ND | ND |
| #178 | - | ND | ND | ND | - | ND | ND |
| #179 | - | ND | ND | ND | - | ND | ND |
| #180 | - | ND | ND | ND | - | ND | ND |
| #181 | - | ND | ND | ND | - | ND | ND |
| #182 | - | ND | ND | ND | - | ND | ND |
| #183 | - | ND | ND | ND | - | ND | ND |
| #184 | - | ND | ND | ND | - | ND | ND |
| #185 | - | ND | ND | ND | - | ND | ND |
| #186 | - | ND | ND | ND | - | ND | ND |
| #187 | - | ND | ND | ND | - | ND | ND |
| #188 | - | ND | ND | ND | - | ND | ND |
| #189 | - | ND | ND | ND | - | ND | ND |
| #190 | - | ND | ND | ND | - | ND | ND |
| #191 | - | ND | ND | ND | - | ND | ND |
| #192 | - | ND | ND | ND | - | ND | ND |
| #193 | - | ND | ND | ND | - | ND | ND |
| #194 | - | ND | ND | ND | - | ND | ND |
| #195 | - | ND | ND | ND | - | ND | ND |
| #196 | - | ND | ND | ND | - | ND | ND |
| #197 | - | ND | ND | ND | - | ND | ND |
| #198 | - | ND | ND | ND | - | ND | ND |
| #199 | - | ND | ND | ND | - | ND | ND |
| #200 | - | ND | ND | ND | - | ND | ND |
| #201 | - | ND | ND | ND | - | ND | ND |
| #202 | - | ND | ND | ND | - | ND | ND |
| #203 | - | ND | ND | ND | - | ND | ND |
| #204 | - | ND | ND | ND | - | ND | ND |
| #205 | - | ND | ND | ND | - | ND | ND |
| #206 | - | ND | ND | ND | - | ND | ND |
| #207 | - | ND | ND | ND | - | ND | ND |
| #208 | - | ND | ND | ND | - | ND | ND |
| #209 | - | ND | ND | ND | - | ND | ND |
| #210 | - | ND | ND | ND | - | ND | ND |
| #211 | - | ND | ND | ND | - | ND | ND |
| #212 | - | ND | ND | ND | - | ND | ND |
| #213 | - | ND | ND | ND | - | ND | ND |
| #214 | - | ND | ND | ND | - | ND | ND |
| #215 | - | ND | ND | ND | - | ND | ND |
| #216 | - | ND | ND | ND | - | ND | ND |
| #217 | - | ND | ND | ND | - | ND | ND |
| #218 | - | ND | ND | ND | - | ND | ND |
| #219 | - | ND | ND | ND | - | ND | ND |
| #220 | - | ND | ND | ND | - | ND | ND |
| #221 | - | ND | ND | ND | - | ND | ND |
| #222 | - | ND | ND | ND | - | ND | ND |
| #223 | - | ND | ND | ND | - | ND | ND |
| #224 | - | ND | ND | ND | - | ND | ND |
| #225 | - | ND | ND | ND | - | ND | ND |
| #226 | - | ND | ND | ND | - | ND | ND |
| #227 | - | ND | ND | ND | - | ND | ND |
| #228 | - | ND | ND | ND | - | ND | ND |
| #229 | - | ND | ND | ND | - | ND | ND |
| #230 | - | ND | ND | ND | - | ND | ND |
| #231 | - | ND | ND | ND | - | ND | ND |
| #232 | - | ND | ND | ND | - | ND | ND |
| #233 | - | ND | ND | ND | - | ND | ND |
| #234 | - | ND | ND | ND | - | ND | ND |
| #235 | - | ND | ND | ND | - | ND | ND |
| #236 | - | ND | ND | ND | - | ND | ND |
| #237 | - | ND | ND | ND | - | ND | ND |
| #238 | - | ND | ND | ND | - | ND | ND |
| #239 | - | ND | ND | ND | - | ND | ND |
| #240 | - | ND | ND | ND | - | ND | ND |
| #241 | - | ND | ND | ND | - | ND | ND |
| #242 | - | ND | ND | ND | - | ND | ND |
| #243 | - | ND | ND | ND | - | ND | ND |
| #244 | - | ND | ND | ND | - | ND | ND |
| #245 | - | ND | ND | ND | - | ND | ND |
| #246 | - | ND | ND | ND | - | ND | ND |
| #247 | - | ND | ND | ND | - | ND | ND |
| #248 | - | ND | ND | ND | - | ND | ND |
| #249 | - | ND | ND | ND | - | ND | ND |
| #250 | - | ND | ND | ND | - | ND | ND |
| #251 | - | ND | ND | ND | - | ND | ND |
| #252 | - | ND | ND | ND | - | ND | ND |
| #253 | - | ND | ND | ND | - | ND | ND |
| #254 | - | ND | ND | ND | - | ND | ND |
| #255 | - | ND | ND | ND | - | ND | ND |
| #256 | - | ND | ND | ND | - | ND | ND |
| #257 | - | ND | ND | ND | - | ND | ND |
| #258 | - | ND | ND | ND | - | ND | ND |
| #259 | - | ND | ND | ND | - | ND | ND |
| #260 | - | ND | ND | ND | - | ND | ND |
| #261 | - | ND | ND | ND | - | ND | ND |
| #262 | - | ND | ND | ND | - | ND | ND |
| #263 | - | ND | ND | ND | - | ND | ND |
| #264 | - | ND | ND | ND | - | ND | ND |
| #265 | - | ND | ND | ND | - | ND | ND |
| #266 | - | ND | ND | ND | - | ND | ND |
| #267 | - | ND | ND | ND | - | ND | ND |
| #268 | - | ND | ND | ND | - | ND | ND |
| #269 | - | ND | ND | ND | - | ND | ND |
| #270 | - | ND | ND | ND | - | ND | ND |
| #271 | - | ND | ND | ND | - | ND | ND |
| #272 | - | ND | ND | ND | - | ND | ND |
| #273 | - | ND | ND | ND | - | ND | ND |
| #274 | - | ND | ND | ND | - | ND | ND |
| #275 | - | ND | ND | ND | - | ND | ND |
| #276 | - | ND | ND | ND | - | ND | ND |
| #277 | - | ND | ND | ND | - | ND | ND |
| #278 | - | ND | ND | ND | - | ND | ND |
| #279 | - | ND | ND | ND | - | ND | ND |
| #280 | - | ND | ND | ND | - | ND | ND |
| #281 | - | ND | ND | ND | - | ND | ND |
| #282 | - | ND | ND | ND | - | ND | ND |
| #283 | - | ND | ND | ND | - | ND | ND |
| #284 | - | ND | ND | ND | - | ND | ND |
| #285 | - | ND | ND | ND | - | ND | ND |
| #286 | - | ND | ND | ND | - | ND | ND |
| #287 | - | ND | ND | ND | - | ND | ND |
| #288 | - | ND | ND | ND | - | ND | ND |
| #289 | - | ND | ND | ND | - | ND | ND |
| #290 | - | ND | ND | ND | - | ND | ND |
| #291 | - | ND | ND | ND | - | ND | ND |
| #292 | - | ND | ND | ND | - | ND | ND |
| #293 | - | ND | ND | ND | - | ND | ND |
| #294 | - | ND | ND | ND | - | ND | ND |
| #295 | - | ND | ND | ND | - | ND | ND |
| #296 | - | ND | ND | ND | - | ND | ND |
| #297 | - | ND | ND | ND | - | ND | ND |
| #298 | - | ND | ND | ND | - | ND | ND |
| #299 | - | ND | ND | ND | - | ND | ND |
| #300 | - | ND | ND | ND | - | ND | ND |
| #301 | - | ND | ND | ND | - | ND | ND |
| #302 | - | ND | ND | ND | - | ND | ND |
| #303 | - | ND | ND | ND | - | ND | ND |
| #304 | - | ND | ND | ND | - | ND | ND |
| #305 | - | ND | ND | ND | - | ND | ND |
| #306 | - | ND | ND | ND | - | ND | ND |
| #307 | - | ND | ND | ND | - | ND | ND |
| #308 | - | ND | ND | ND | - | ND | ND |
| #309 | - | ND | ND | ND | - | ND | ND |
| #310 | - | ND | ND | ND | - | ND | ND |
| #311 | - | ND | ND | ND | - | ND | ND |
| #312 | - | ND | ND | ND | - | ND | ND |
| #313 | - | ND | ND | ND | - | ND | ND |
| #314 | - | ND | ND | ND | - | ND | ND |
| #315 | - | ND | ND | ND | - | ND | ND |
| #316 | - | ND | ND | ND | - | ND | ND |
| #317 | - | ND | ND | ND | - | ND | ND |
| #318 | - | ND | ND | ND | - | ND | ND |
| #319 | - | ND | ND | ND | - | ND | ND |
| #320 | - | ND | ND | ND | - | ND | ND |
| #321 | - | ND | ND | ND | - | ND | ND |
| #322 | - | ND | ND | ND | - | ND | ND |
| #323 | - | ND | ND | ND | - | ND | ND |
| #324 | - | ND | ND | ND | - | ND | ND |
| #325 | - | ND | ND | ND | - | ND | ND |
| #326 | - | ND | ND | ND | - | ND | ND |
| #327 | - | ND | ND | ND | - | ND | ND |
| #328 | - | ND | ND | ND | - | ND | ND |
| #329 | - | ND | ND | ND | - | ND | ND |
| #330 | - | ND | ND | ND | - | ND | ND |
| #331 | - | ND | ND | ND | - | ND | ND |
| #332 | - | ND | ND | ND | - | ND | ND |
| #333 | - | ND | ND | ND | - | ND | ND |
| #334 | - | ND | ND | ND | - | ND | ND |
| #335 | - | ND | ND | ND | - | ND | ND |
| #336 | - | ND | ND | ND | - | ND | ND |
| #337 | - | ND | ND | ND | - | ND | ND |
| #338 | - | ND | ND | ND | - | ND | ND |
| #339 | - | ND | ND | ND | - | ND | ND |
| #340 | - | ND | ND | ND | - | ND | ND |
| #341 | - | ND | ND | ND | - | ND | ND |
| #342 | - | ND | ND | ND | - | ND | ND |
| #343 | - | ND | ND | ND | - | ND | ND |
| #344 | - | ND | ND | ND | - | ND | ND |
| #345 | - | ND | ND | ND | - | ND | ND |
| #346 | - | ND | ND | ND | - | ND | ND |
| #347 | - | ND | ND | ND | - | ND | ND |
| #348 | - | ND | ND | ND | - | ND | ND |
| #349 | - | ND | ND | ND | - | ND | ND |
| #350 | - | ND | ND | ND | - | ND | ND |
| #351 | - | ND | ND | ND | - | ND | ND |
| #352 | - | ND | ND | ND | - | ND | ND |
| #333 | - | ND | ND | ND | - | ND | ND |
| #334 | - | ND | ND | ND | - | ND | ND |
| #335 | - | ND | ND | ND | - | ND | ND |
| #336 | - | ND | ND | ND | - | ND | ND |
| #337 | - | ND | ND | ND | - | ND | ND |
| #338 | - | ND | ND | ND | - | ND | ND |
| #339 | - | ND | ND | ND | - | ND | ND |
| #340 | - | ND | ND | ND | - | ND | ND |
| #341 | - | ND | ND | ND | - | ND | ND |
| #342 | - | ND | ND | ND | - | ND | ND |
| #343 | - | ND | ND | ND | - | ND | ND |
| #344 | - | ND | ND | ND | - | ND | ND |
| #345 | - | ND | ND | ND | - | ND | ND |
| #346 | - | ND | ND | ND | - | ND | ND |
| #347 | - | ND | ND | ND | - | ND | ND |
| #348 | - | ND | ND | ND | - | ND | ND |
| #349 | - | ND | ND | ND | - | ND | ND |
| #350 | - | ND | ND | ND | - | ND | ND |
| #351 | - | ND | ND | ND | - | ND | ND |
| #352 | - | ND | ND | ND | - | ND | ND |
| #353 | - | ND | ND | ND | - | ND | ND |
| #354 | - | ND | ND | ND | - | ND | ND |
| #355 | - | ND | ND | ND | - | ND | ND |
| #356 | - | ND | ND | ND | - | ND | ND |
| #357 | - | ND | ND | ND | - | ND | ND |
| #358 | - | ND | ND | ND | - | ND | ND |
| #359 | - | ND | ND | ND | - | ND | ND |

*Ct* cycle threshold, *ND* not detected, *NIID* National Institute of Infectious Diseases, *RT-PCR* reverse transcription polymerase chain reaction.
