# Supplementary material for: The evaluation of the utility of the GENECUBE HQ SARS-CoV-2 for anterior nasal samples and saliva samples with a new rapid examination protocol
Source: PLoS One. 2021 Dec 31;16(12):e0262159. doi: 10.1371/journal.pone.0262159 (PMC8719657; doi:10.1371/journal.pone.0262159)
Supplement: S2 Table — (DOCX) [file pone.0262159.s003.docx]

**S2 Table. Results of SARS-Cov-2 detection for saliva samples.**

| Saliva samples | | | | | | Nasopharyngeal samples collected from the same patient simultaneously | | |
| --- | --- | --- | --- | --- | --- | --- | --- | --- |
| Sample No. | GENECUBE_®_  (Standard method with magLEAD) | GENECUBE_®_  (Rapid method with magLEAD) | Real-time RT-PCR  (N2 NIID method) | | | GENECUBE_®_  (Standard method with magLEAD) | Real-time RT-PCR  (N2 NIID method) | |
|  |  |  | Ct | Ct | Copies/test |  | Ct | Copies/test |
| #1 | + | + | 18.7 | 19.0 | 113,330 | + | 12.3 | 56,410,000 |
| #2 | + | + | 14.5 | 14.5 | 1,311,050 | + | 14.2 | 4,314,000 |
| #3 | + | + | 32.5 | 32.3 | 51 | + | 23.7 | 6,357 |
| #4 | + | + | 21.8 | 22.0 | 19,160 | + | 14.8 | 2,534,000 |
| #5 | + | + | 23.2 | 23.3 | 9,013 | + | 20.2 | 87,800 |
| #6 | + | + | 30.8 | 30.9 | 121 | + | 19.2 | 123,600 |
| #7 | + | + | 35.2 | ND | 8 | + | 29.4 | 238 |
| #8 | + | + | 15.6 | 15.7 | 694,100 | + | 10.7 | 14,010,000 |
| #9 | + | + | 22.4 | 21.9 | 16,485 | + | 18.3 | 217,800 |
| #10 | + | + | 31.9 | 31.9 | 68 | + | 22.9 | 11,010 |
| #11 | - | + | ND | ND | ND | + | 20.1 | 71,130 |
| #12 | + | + | 14.3 | 14.5 | 1,417,500 | + | 14.9 | 960,000 |
| #13 | + | + | 15.8 | 15.9 | 623,700 | + | 14.7 | 3,024,000 |
| #14 | + | + | 31.1 | ND | 87 | + | 18.6 | 253,200 |
| #15 | + | + | 22.7 | 22.2 | 13,780 | + | 15.5 | 5,599,000 |
| #16 | + | + | 25.4 | 25.0 | 2,952 | + | 15.1 | 2,382,000 |
| #17 | - | - | ND | ND | ND | + | 29.7 | 172 |
| #18 | + | + | 28.9 | 28.9 | 359 | + | 23.6 | 6,551 |
| #19 | + | + | 24.3 | 24.6 | 4,578 | + | 27.9 | 548 |
| #20 | + | + | 14.6 | 14.6 | 1,257,850 | + | 16.8 | 546,900 |
| #21 | + | + | 28.3 | 28.4 | 498 | + | 30.4 | 120 |
| #22 | + | + | 19.7 | 20.0 | 63,540 | + | 15.3 | 1,477,000 |
| #23 | + | + | 32.7 | 33.1 | 38 | + | 20.7 | 32,500 |
| #24 | + | + | 27.2 | 27.3 | 915 | + | 21.2 | 46,920 |
| #25 | + | + | 16.9 | 16.9 | 334,250 | + | 11.2 | 6,972,000 |
| #26 | + | + | 28.5 | 29.0 | 401 | + | 17.1 | 646,000 |
| #27 | + | + | 30.4 | 30.3 | 162 | + | 17.1 | 366,800 |
| #28 | + | + | 29.1 | 28.9 | 340 | + | 21.2 | 35,070 |
| #29 | + | + | 24.7 | 24.9 | 3,771 | + | 15.8 | 991,000 |
| #30 | + | + | 20.0 | 20.2 | 54,105 | + | 14.1 | 15,390,000 |
| #31 | + | + | ND | ND | ND | + | 23.2 | 7,789 |
| #32 | + | + | 26.1 | 26.3 | 1,672 | + | 19.4 | 152,000 |
| #33 | + | + | 21.0 | 20.5 | 37,410 | + | 17.1 | 671,500 |
| #34 | + | + | 15.0 | 15.1 | 959,300 | + | 18.1 | 148,800 |
| #35 | + | + | 20.8 | 21.0 | 34,915 | + | 16.5 | 379,100 |
| #36 | + | + | 19.9 | 19.3 | 69,525 | + | 15.6 | 1,236,000 |
| #37 | + | + | 23.0 | 23.2 | 9,915 | + | 19.7 | 248,600 |
| #38 | + | + | 29.0 | 28.6 | 388 | + | 25.7 | 1,782 |
| #39 | - | + | ND | ND | ND | + | 33.9 | 16 |
| #40 | + | + | 25.4 | 25.2 | 2,784 | + | 15.0 | 1,073,000 |
| #41 | + | + | 23.2 | 23.6 | 8,317 | + | 24.9 | 3,272 |
| #42 | + | + | 22.7 | 23.0 | 11,610 | + | 13.7 | 3,828,000 |
| #43 | + | + | 25.7 | 25.3 | 2,482 | + | 17.0 | 1,878,000 |
| #44 | + | + | 27.2 | 27.5 | 877 | + | 33.2 | 19 |
| #45 | + | + | 27.4 | 27.4 | 865 | + | 20.7 | 32,690 |
| #46 | + | + | 17.9 | 18.0 | 184,300 | + | 14.1 | 1,510,000 |
| #47 | + | + | 26.3 | 26.4 | 1,542 | + | 13.5 | 2,127,000 |
| #48 | + | + | 17.0 | 17.1 | 310,350 | + | 16.0 | 1,195,000 |
| #49 | + | + | 23.3 | 23.3 | 8,728 | + | 13.5 | 24,140,000 |
| #50 | + | + | 24.2 | 24.5 | 4,800 | + | 20.8 | 39,270 |
| #51 | + | + | 22.3 | 22.6 | 14,150 | + | 16.3 | 1,062,000 |
| #52 | + | + | 25.1 | 25.3 | 2,993 | + | 17.7 | 441,300 |
| #53 | + | + | 27.7 | 27.5 | 747 | + | 20.3 | 156,200 |
| #54 | + | + | 20.7 | 21.1 | 35,410 | + | 21.7 | 57,670 |
| #55 | + | + | 27.3 | 27.5 | 870 | + | 18.1 | 178,000 |
| #56 | + | + | 34.4 | ND | 12 | + | 21.2 | 41,280 |
| #57 | + | + | 22.0 | 22.3 | 17,005 | + | 31.4 | 64 |
| #58 | + | + | 30.0 | 30.5 | 173 | + | 17.8 | 240,400 |
| #59 | + | + | 18.4 | 18.6 | 137,800 | + | 13.5 | 4,642,000 |
| #60 | + | + | 14.8 | 14.9 | 1,072,400 | + | 14.7 | 2,772,000 |
| #61 | - | - | ND | ND | ND |  |  |  |
| #62 | - | - | ND | ND | ND |  |  |  |
| #63 | - | - | ND | ND | ND |  |  |  |
| #64 | - | - | ND | ND | ND |  |  |  |
| #65 | - | - | ND | ND | ND |  |  |  |
| #66 | - | - | ND | ND | ND |  |  |  |
| #67 | - | - | ND | ND | ND |  |  |  |
| #68 | - | - | ND | ND | ND |  |  |  |
| #69 | - | - | ND | ND | ND |  |  |  |
| #70 | - | - | ND | ND | ND |  |  |  |
| #71 | - | - | ND | ND | ND |  |  |  |
| #72 | - | - | ND | ND | ND |  |  |  |
| #73 | - | - | ND | ND | ND |  |  |  |
| #74 | - | - | ND | ND | ND |  |  |  |
| #75 | - | - | ND | ND | ND |  |  |  |
| #76 | - | - | ND | ND | ND |  |  |  |
| #77 | - | - | ND | ND | ND |  |  |  |
| #78 | - | - | ND | ND | ND |  |  |  |
| #79 | - | - | ND | ND | ND |  |  |  |
| #80 | - | - | ND | ND | ND |  |  |  |
| #81 | - | - | ND | ND | ND |  |  |  |
| #82 | - | - | ND | ND | ND |  |  |  |
| #83 | - | - | ND | ND | ND |  |  |  |
| #84 | - | - | ND | ND | ND |  |  |  |
| #85 | - | - | ND | ND | ND |  |  |  |
| #86 | - | - | ND | ND | ND |  |  |  |
| #87 | - | - | ND | ND | ND |  |  |  |
| #88 | - | - | ND | ND | ND |  |  |  |
| #89 | - | - | ND | ND | ND |  |  |  |
| #90 | - | - | ND | ND | ND |  |  |  |
| #91 | - | - | ND | ND | ND |  |  |  |
| #92 | - | - | ND | ND | ND |  |  |  |
| #93 | - | - | ND | ND | ND |  |  |  |
| #94 | - | - | ND | ND | ND |  |  |  |
| #95 | - | - | ND | ND | ND |  |  |  |
| #96 | - | - | ND | ND | ND |  |  |  |
| #97 | - | - | ND | ND | ND |  |  |  |
| #98 | - | - | ND | ND | ND |  |  |  |
| #99 | - | - | ND | ND | ND |  |  |  |
| #100 | - | - | ND | ND | ND |  |  |  |
| #101 | - | - | ND | ND | ND |  |  |  |
| #102 | - | - | ND | ND | ND |  |  |  |
| #103 | - | - | ND | ND | ND |  |  |  |
| #104 | - | - | ND | ND | ND |  |  |  |
| #105 | - | - | ND | ND | ND |  |  |  |
| #106 | - | - | ND | ND | ND |  |  |  |
| #107 | - | - | ND | ND | ND |  |  |  |
| #108 | - | - | ND | ND | ND |  |  |  |
| #109 | - | - | ND | ND | ND |  |  |  |
| #110 | - | - | ND | ND | ND |  |  |  |
| #111 | - | - | ND | ND | ND |  |  |  |
| #112 | - | - | ND | ND | ND |  |  |  |
| #113 | - | - | ND | ND | ND |  |  |  |
| #114 | - | - | ND | ND | ND |  |  |  |
| #115 | - | - | ND | ND | ND |  |  |  |
| #116 | - | - | ND | ND | ND |  |  |  |
| #117 | - | - | ND | ND | ND |  |  |  |
| #118 | - | - | ND | ND | ND |  |  |  |
| #119 | - | - | ND | ND | ND |  |  |  |
| #120 | - | - | ND | ND | ND |  |  |  |
| #121 | - | - | ND | ND | ND |  |  |  |
| #122 | - | - | ND | ND | ND |  |  |  |
| #123 | - | - | ND | ND | ND |  |  |  |
| #124 | - | - | ND | ND | ND |  |  |  |
| #125 | - | - | ND | ND | ND |  |  |  |
| #126 | - | - | ND | ND | ND |  |  |  |
| #127 | - | - | ND | ND | ND |  |  |  |
| #128 | - | - | ND | ND | ND |  |  |  |
| #129 | - | - | ND | ND | ND |  |  |  |
| #130 | - | - | ND | ND | ND |  |  |  |
| #131 | - | - | ND | ND | ND |  |  |  |
| #132 | - | - | ND | ND | ND |  |  |  |
| #133 | - | - | ND | ND | ND |  |  |  |
| #134 | - | - | ND | ND | ND |  |  |  |
| #135 | - | - | ND | ND | ND |  |  |  |
| #136 | - | - | ND | ND | ND |  |  |  |
| #137 | - | - | ND | ND | ND |  |  |  |
| #138 | - | - | ND | ND | ND |  |  |  |
| #139 | - | - | ND | ND | ND |  |  |  |
| #140 | - | - | ND | ND | ND |  |  |  |
| #141 | - | - | ND | ND | ND |  |  |  |
| #142 | - | - | ND | ND | ND |  |  |  |
| #143 | - | - | ND | ND | ND |  |  |  |
| #144 | - | - | ND | ND | ND |  |  |  |
| #145 | - | - | ND | ND | ND |  |  |  |
| #146 | - | - | ND | ND | ND |  |  |  |
| #147 | - | - | ND | ND | ND |  |  |  |
| #148 | - | - | ND | ND | ND |  |  |  |
| #149 | - | - | ND | ND | ND |  |  |  |
| #150 | - | - | ND | ND | ND |  |  |  |
| #151 | - | - | ND | ND | ND |  |  |  |
| #152 | - | - | ND | ND | ND |  |  |  |
| #153 | - | - | ND | ND | ND |  |  |  |
| #154 | - | - | ND | ND | ND |  |  |  |
| #155 | - | - | ND | ND | ND |  |  |  |
| #156 | - | - | ND | ND | ND |  |  |  |
| #157 | - | - | ND | ND | ND |  |  |  |
| #158 | - | - | ND | ND | ND |  |  |  |
| #159 | - | - | ND | ND | ND |  |  |  |
| #160 | - | - | ND | ND | ND |  |  |  |
| #161 | - | - | ND | ND | ND |  |  |  |
| #162 | - | - | ND | ND | ND |  |  |  |
| #163 | - | - | ND | ND | ND |  |  |  |
| #164 | - | - | ND | ND | ND |  |  |  |
| #165 | - | - | ND | ND | ND |  |  |  |
| #166 | - | - | ND | ND | ND |  |  |  |
| #167 | - | - | ND | ND | ND |  |  |  |
| #168 | - | - | ND | ND | ND |  |  |  |
| #169 | - | - | ND | ND | ND |  |  |  |
| #170 | - | - | ND | ND | ND |  |  |  |
| #171 | - | - | ND | ND | ND |  |  |  |
| #172 | - | - | ND | ND | ND |  |  |  |
| #173 | - | - | ND | ND | ND |  |  |  |
| #174 | - | - | ND | ND | ND |  |  |  |
| #175 | - | - | ND | ND | ND |  |  |  |
| #176 | - | - | ND | ND | ND |  |  |  |
| #177 | - | - | ND | ND | ND |  |  |  |
| #178 | - | - | ND | ND | ND |  |  |  |
| #179 | - | - | ND | ND | ND |  |  |  |
| #180 | - | - | ND | ND | ND |  |  |  |
| #181 | - | - | ND | ND | ND |  |  |  |
| #182 | - | - | ND | ND | ND |  |  |  |
| #183 | - | - | ND | ND | ND |  |  |  |
| #184 | - | - | ND | ND | ND |  |  |  |
| #185 | - | - | ND | ND | ND |  |  |  |
| #186 | - | - | ND | ND | ND |  |  |  |
| #187 | - | - | ND | ND | ND |  |  |  |
| #188 | - | - | ND | ND | ND |  |  |  |
| #189 | - | - | ND | ND | ND |  |  |  |
| #190 | - | - | ND | ND | ND |  |  |  |
| #191 | - | - | ND | ND | ND |  |  |  |
| #192 | - | - | ND | ND | ND |  |  |  |
| #193 | - | - | ND | ND | ND |  |  |  |
| #194 | - | - | ND | ND | ND |  |  |  |
| #195 | - | - | ND | ND | ND |  |  |  |
| #196 | - | - | ND | ND | ND |  |  |  |
| #197 | - | - | ND | ND | ND |  |  |  |
| #198 | - | - | ND | ND | ND |  |  |  |
| #199 | - | - | ND | ND | ND |  |  |  |
| #200 | - | - | ND | ND | ND |  |  |  |
| #201 | - | - | ND | ND | ND |  |  |  |
| #202 | - | - | ND | ND | ND |  |  |  |
| #203 | - | - | ND | ND | ND |  |  |  |
| #204 | - | - | ND | ND | ND |  |  |  |
| #205 | - | - | ND | ND | ND |  |  |  |
| #206 | - | - | ND | ND | ND |  |  |  |
| #207 | - | - | ND | ND | ND |  |  |  |
| #208 | - | - | ND | ND | ND |  |  |  |
| #209 | - | - | ND | ND | ND |  |  |  |
| #210 | - | - | ND | ND | ND |  |  |  |
| #211 | - | - | ND | ND | ND |  |  |  |
| #212 | - | - | ND | ND | ND |  |  |  |
| #213 | - | - | ND | ND | ND |  |  |  |
| #214 | - | - | ND | ND | ND |  |  |  |
| #215 | - | - | ND | ND | ND |  |  |  |
| #216 | - | - | ND | ND | ND |  |  |  |
| #217 | - | - | ND | ND | ND |  |  |  |
| #218 | - | - | ND | ND | ND |  |  |  |
| #219 | - | - | ND | ND | ND |  |  |  |
| #220 | - | - | ND | ND | ND |  |  |  |
| #221 | - | - | ND | ND | ND |  |  |  |
| #222 | - | - | ND | ND | ND |  |  |  |
| #223 | - | - | ND | ND | ND |  |  |  |
| #224 | - | - | ND | ND | ND |  |  |  |
| #225 | - | - | ND | ND | ND |  |  |  |
| #226 | - | - | ND | ND | ND |  |  |  |
| #227 | - | - | ND | ND | ND |  |  |  |
| #228 | - | - | ND | ND | ND |  |  |  |
| #229 | - | - | ND | ND | ND |  |  |  |
| #230 | - | - | ND | ND | ND |  |  |  |
| #231 | - | - | ND | ND | ND |  |  |  |
| #232 | - | - | ND | ND | ND |  |  |  |
| #233 | - | - | ND | ND | ND |  |  |  |
| #234 | - | - | ND | ND | ND |  |  |  |
| #235 | - | - | ND | ND | ND |  |  |  |
| #236 | - | - | ND | ND | ND |  |  |  |
| #237 | - | - | ND | ND | ND |  |  |  |
| #238 | - | - | ND | ND | ND |  |  |  |
| #239 | - | - | ND | ND | ND |  |  |  |
| #240 | - | - | ND | ND | ND |  |  |  |

*Ct* cycle threshold, *ND* not detected, *NIID* National Institute of Infectious Diseases, *RT-PCR* reverse transcription polymerase chain reaction
