# Supplementary material for: The evaluation of the utility of the GENECUBE HQ SARS-CoV-2 for anterior nasal samples and saliva samples with a new rapid examination protocol
Source: PLoS One. 2021 Dec 31;16(12):e0262159. doi: 10.1371/journal.pone.0262159 (PMC8719657; doi:10.1371/journal.pone.0262159)
Supplement: S3 Table — (DOCX) [file pone.0262159.s004.docx]

**S3 Table. Detailed data for the three cases with discordant findings between the three SARS-CoV-2 detection methods for saliva samples*.**

| Sample No. | GENECUBE^®^ | | Real-time RT-PCR  (N2 NIID method) | Real-time RT-PCR  (Roche LightMix Modular SARS and Wuhan CoV E-gene) | | |
| --- | --- | --- | --- | --- | --- | --- |
|  | Standard method  with magLEAD | Rapid method  with magLEAD | Standard method  with magLEAD | Standard method  with magLEAD | Rapid method  with magLEAD | QIAamp^®^ Viral RNA  Mini Kit |
| #11 | – | + | 0/2 (0) | 0/2 (0) | 4/8 (50) | 0/8 (0) |
| #31 | + | + | 0/2 (0) | 2/2 (100) | NA | NA |
| #39 | – | + | 0/2 (0) | 0/2 (0) | 1/8 (12.5) | 0/8 (0) |
| + positive, *−* negative, *NIID* National Institute of Infectious Diseases, *RT-PCR*, reverse transcription polymerase chain reaction, *NA*, Not applicable | | | | | | |
| Values indicate N of detection/N of test (detection rate). | | | | | | |
| All of three saliva samples were obtained from COVID-19 patients (supplementary table 2) | | | | | | |
| For #11, #39, additional RT-PCR (E-gene) was performed with purified samples with rapid method with magLEAD and QIAamp^®^ Viral RNA Mini Kit, because RT-PCR (E-gene) analysis was negative with purified samples with Standard method with magLEAD. | | | | | | |
