# Supplementary material for: The evaluation of the utility of the GENECUBE HQ SARS-CoV-2 for anterior nasal samples and saliva samples with a new rapid examination protocol
Source: PLoS One. 2021 Dec 31;16(12):e0262159. doi: 10.1371/journal.pone.0262159 (PMC8719657; doi:10.1371/journal.pone.0262159)
Supplement: Attachment — Submitted filename: Response to reviewers.docx [file pone.0262159.s006.docx]

***Suggestion****: Thank you for the changes to the manuscript, which improves the clarity of study. One minor suggestion I would have is to add a limitation about the lack of low viral load samples tested in the evaluation. line 202,* *only 3 samples were CT >30. This is a small proportion of your positives and that could have improved concordance as too few were near the limit of detection.*

**Response**: Thank you very much for your suggestion. As you pointed out, the proportion of low viral load samples (Ct ≥ 30) was small for the evaluation of the GENECUBE® examination using anterior nasal samples, which could have improved thus concordance rates of the current study. We therefore added this point as one limitation associated with our study in the limitation section of the discussion.
